# Enhanced spectro-temporal feature extraction for prosthetic control using variational mode decomposition

**DOI:** 10.1038/s41598-025-28156-6

**Published:** 2025-11-22

**Authors:** Uzma Shafiq, Asim Waris, Faisal Amin, Ajdar Ullah, Hassan Ashraf, Muhammad Jawad Khan, Muhammad Adeel Ijaz, Fawwaz Hazzazi, Khalid Ansari, Syed Omer Gilani

**Affiliations:** 1 Department of Biomedical Engineering and Sciences, School of Mechanical and Manufacturing Engineering, National University of Sciences and Technology (NUST), Islamabad, Pakistan; 2 Laboratory of Movement Analysis (LAM-Motion Lab), University of Liège, Liège, Belgium; 3 Department of Electrical Engineering, College of Engineering, Prince Sattam bin Abdulaziz University, Al-Kharj, 11942 Saudi Arabia; 4 Faculty of Medical and Health Sciences, Liwa University, Abu Dhabi, United Arab Emirates; 5 Department of Electrical, Computer, and Biomedical Engineering, Abu Dhabi University, Abu Dhabi, United Arab Emirates

**Keywords:** Electromyography, Rehabilitation, Variational mode decomposition, Pattern recognition, Spectro-temporal, Biomedical engineering, Engineering

## Abstract

Rehabilitation systems play a vital role in improving the lifestyle of amputees or individuals with congenitally deficient limbs. However, the currently used pattern recognition techniques for different rehabilitation systems are highly noise sensitive. Inadequate feature extraction results in difficulty distinguishing similar gestures. Moreover, the implementation of previously proposed techniques on amputees is minimal. To address the issue of high sensitivity to noise, this study proposes a new spectro-temporal feature set for the enhanced implementation of rehabilitation systems. The efficacy of the proposed feature extraction technique was evaluated by comparing it with four other features aimed at addressing the broader applicability aspect. The Variational Mode Decomposition (VMD) of electromyography signals decomposes the signal into multiple variational mode functions (VMFs). A singular value representation is obtained from each VMF with a distinct spectral band using Singular Value Decomposition (SVD). The feature vectors employed consisted of a time-domain feature set, two spectro-temporal feature sets utilizing VMD and Empirical Mode Decomposition (EMD), and two combined feature sets. The results indicated that the proposed feature extraction technique outperforms the remaining feature sets with an accuracy of 97% for Dataset I, which comprises EMG recordings from 10 healthy subjects performing four hand and wrist motions. Statistical analysis revealed overall significance among all feature vectors (p-value < 0.05). The generalizability of the proposed technique was evaluated using Ninapro DB2 for healthy subjects with 49 motions, DB3 for amputees with 52 motions, and DB8 for both healthy and amputee subjects with 9 motions. The accuracies obtained for DB2, DB3, and DB9 were 91.43%, 95.66%, and 98.16% respectively. The analysis for the optimum number of VMFs revealed that 6 VMFs provide a reasonable tradeoff between accuracy and execution time.

**Supplementary Information:**

The online version contains supplementary material available at 10.1038/s41598-025-28156-6.

## Introduction

Electromyography (EMG) signals are the electrical activity generated during muscle contraction. The irregularity in the generated EMG signals can occur due to many genetic or acquired abnormalities, such as muscular dystrophy, nerve damage, and peripheral neuropathy^1–3^. EMG-triggered assistive devices can aid patients with such abnormalities. The basic unit of these devices carries out the detection of EMG signals, processing, and classification for respective applications^4^.

The controllability of the above-mentioned assistive devices depends entirely on the ability to classify the EMG signals. Poor classification in terms of motion recognition results in a system that is limited to laboratory settings and cannot be used in a real-time environment^5^. The classification accuracy of Rehabilitation Systems (RS) depends on the number and placement site of electrodes used to acquire data, the classification algorithm employed, and the extracted EMG features. Improving the accuracy of RS based on these factors has been the prime focus of researchers^6,7^. The already developed RS-based applications include hand rehabilitation^8^, classification of hand movements^9^, and a muscle interface for car drivers^10^. Currently, the primary issue in clinical applications of RS is its robustness. EMG signals are non-stationary and non-stochastic in nature. As the accuracy of RS is highly dependent on the features used for classification, this work aims to introduce features that can incorporate the non-stationarity of the EMG signal. Previously, time-domain features have been widely used in RS^11,12^. The most frequently used time-domain features that provide significant classification accuracy are Mean Absolute Value (MAV), Zero-Crossing (ZC), Waveform Length (WL), Root Mean Square (RMS), Standard Error (SE), Signal-to-Noise Ratio (SNR), Standard Deviation (STD), and Variance (VAR).

The extracted time-domain feature set considers the signal as static. It is computed using the signal’s amplitude and dynamics, resulting in a feature set that is highly susceptible to noise and artifacts^13^. The time-domain feature set fails to incorporate the non-stationary nature of EMG signals^14^. Phinyomark studied frequency domain features and time domain features, and it was concluded that frequency domain features are not good at classifying EMG signals^11^. Frequency domain information can be extracted using the Short Time Fourier Transform (STFT), which processes the data within a fixed window size. Still, determining the optimum window size is challenging due to the non-stationary nature of the EMG signal^15–17^. The new time-domain feature sets from the LibEMG open-source EMG feature extraction library^18^, such as LS4 (MFL, MSR, WAMP, LS) and LS9 (MAV, ZC, SSC, WL, RMS, IAV, DASDV, VAR), have also been evaluated^19^.

Time-frequency analysis, although computationally more expensive, resolves these issues. The wavelet domain analysis considers the EMG signal non-stationary and performs the feature extraction process in the time-frequency domain. Continuous wavelet transform has been utilized to extract features for pattern recognition^20^, prosthesis control^21^, and MECS^22^. The wavelet transform-based feature extraction requires prior knowledge about the signal to select the mother wavelet. The mother wavelet chosen and the optimum number of decomposition levels for feature extraction greatly influence the classification accuracy^13^. As the fundamental wavelet function needs to be selected in advance, the choice of mother wavelet can be challenging as the EMG signals are not only non-stationary but also stochastic in nature. Thus, the wavelet transform fails to incorporate the stochastic nature of EMG signals^23,24^.

To eradicate the issue of choosing the mother wavelet in advance, Huang et al. introduced Empirical Mode Decomposition (EMD)^25^. It is an adaptive technique for signal decomposition and is entirely data dependent. The decomposition is carried out in time domain only, thus making EMD computationally less expensive. EMD decomposes the signal into intrinsic modal functions by sifting out fast dynamics and removing the slower ones by averaging the extrema of the signal using different interpolation techniques and extrema finding^25^. EMD results in mode mixing, making it highly sensitive to noise and sampling. Consequently, this results in poor classification accuracy for noisy signals. Since the success of RS is highly dependent on its classification, accuracy and robustness, a more suitable method needs to be implemented for feature extraction.

Dragomiretskiy and Zosso introduced Variational Mode Decomposition (VMD), which is a fully adaptive and intrinsic signal decomposition method^26^. VMD decomposes the signal into its Variational Mode Functions (VMFs) based on frequencies present in the signal, thus eliminating the issue of modal aliasing. The eradication of modal aliasing decreases the sensitivity of VMD towards noise. Dragomiretskiy and Zosso also illustrated the robustness of VMD for a tri-harmonic signal affected by noise^26^. They utilized a signal containing three harmonic components and compared the performance efficacy of VMD and EMD. The decomposed VMFs have a narrow frequency band, which becomes narrower as the frequency increases. VMD uses a quadratic penalty to change the constrained problem to a non-constrained problem, encouraging the reconstruction fidelity^26^. Thus, it provides a more accurate representation of the original EMG signal. These factors make VMD more robust than previously discussed methods, and therefore, it is a more appropriate choice for RS. VMD decomposes the signal in both the time and frequency domains. Thus, the extracted VMFs provide both spectral and temporal information. Studies have been conducted to extract spectral and temporal features using VMD for neuromuscular disorder detection^27^, physical actions classification^28^, detection of different fatigue conditions^29^, and denoising of EMG signals^30^.

VMD was utilized to classify five finger flexion patterns^31^. The drawback of the study is that it comprises only three subjects and reports an accuracy of 83%. Xiao et al. reported a method for classifying hand movements using VMD and a composite permutation entropy index^32^. They used VMD to decompose the signal into subsequent sub-signals and applied permutation entropy to get the required features. However, the permutation entropy index has been widely used to extract specific fluctuations in nonlinear deterministic systems that are weakly contaminated with noise^33^. EMG signals are stochastic. They are contaminated with noise during the data collection procedure^34^. Introducing a chaotic noise signal to the original EMG signal can result in incorrect recognition, making it unsuitable for real-time signals^35^. Moreover, subject-wise classification was carried out, which does not guarantee the generalizability of the proposed method. Yang et al. utilized multivariate VMD to classify hand movements^36^. The employed classification method was notably comprehensive, involving two distinct classification stages. The first stage identifies the super class using Support Vector Machine (SVM) or Random Forest (RF), whereas the accurate prediction of the performed movement was achieved using CNN^36^. The permutation entropy index has been widely used to extract specific fluctuations in weakly noise-contaminated nonlinear deterministic systems^33^. Moreover, no analysis was reported for an optimum number of modes extracted during the feature extraction stage. VMD has been used along with entropy to classify lower limb movements using a single channel^37^. The proposed method was tested only for four lower limb motions, and its efficacy for a more significant number of motions was not analyzed. Moreover, the study fails to address the method’s performance for amputees.

Due to the drawbacks of using entropy, an alternative method that is more stable and can extract vital information from the sub-signals must be introduced. Singular Value decomposition has been widely used for various applications. SVD results in singular values obtained by the square root of eigenvalues. SVD is a reliable method with a strong mathematical foundation, featuring unique singular values and robust numerical algorithms for stability. Its versatility in handling various matrix types has made it widely used in applications like data compression^38^. Moreover, the previous studies analyzed healthy subjects and did not report any results for amputee subjects. Thus, the study proposes a more stable algorithm for feature extraction utilizing SVD rather than permutation entropy to address the volatile nature of entropy used previously by^32^. A computationally less expensive algorithm is utilized to address the concern of the high computational expense used in^36^. Moreover, a maximum of 50 motions were classified to depict the study’s generalizability.

**Fig. 1:**
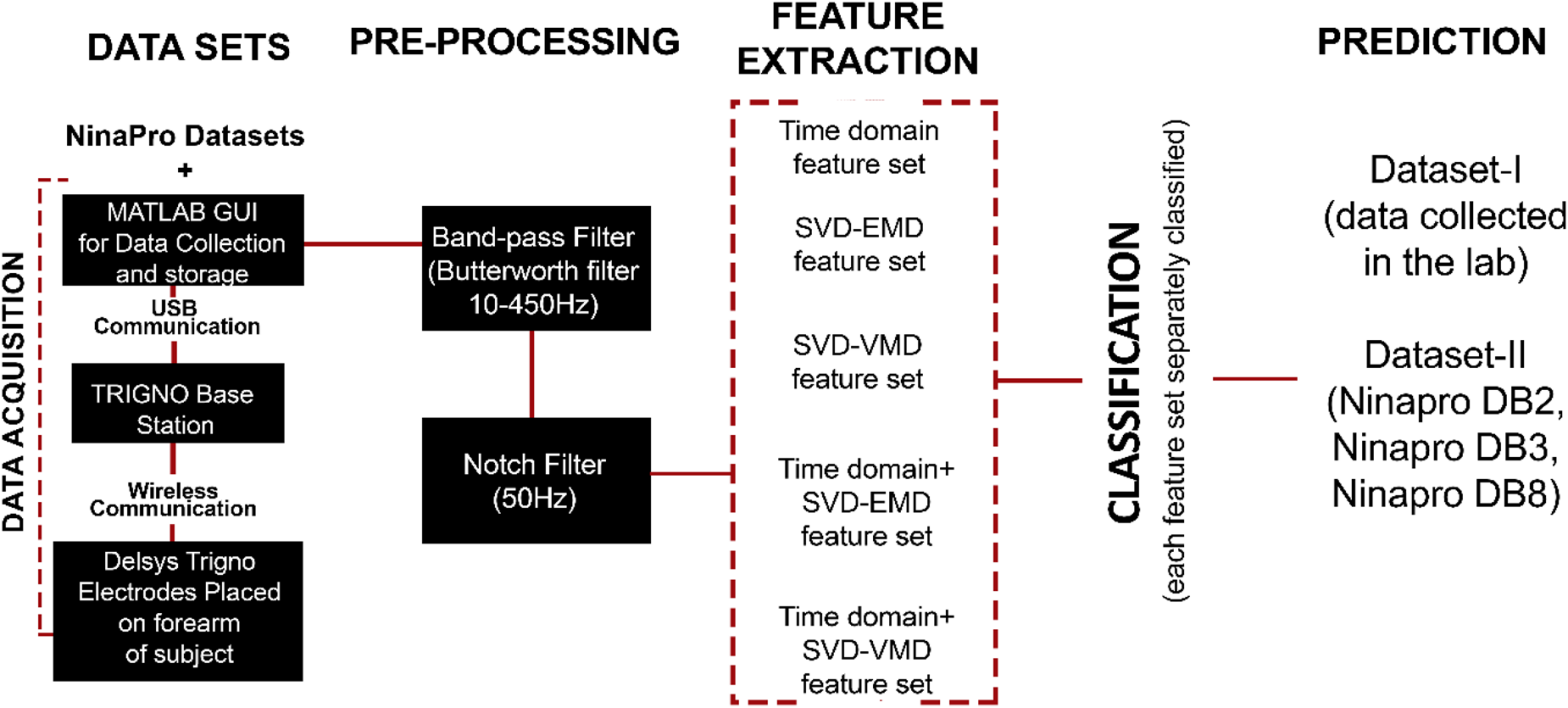
Graphical representation of the proposed method.

The paper compares five formulated feature sets for RS to demonstrate the efficacy of the proposed feature set. Two feature sets were obtained using the decomposition techniques EMD and VMD, along with SVD, resulting in the EMD-SVD and VMD-SVD feature sets. Figure 1 illustrates the steps involved in the study in a graphical representation.

## Methods

### Datasets

Informed consent was obtained from all the subjects before the start of the experiment. All the subjects were adults and provided their written consent. The data collection was conducted in accordance with the relevant guidelines and regulations. Ethical approval for this study was obtained under Approval No.: NUST/SMME-BME/REC/000424/20,122,021 from the National University of Sciences and Technology ethical committee. The data collection process lasted two weeks, from January 7, 2022, to January 21, 2022.

Data from 10 healthy (7 males and 3 females) subjects between 20 and 30 years (24.7 ± 2.2) were collected. No injuries were observed in any of the subjects at the time of data collection. Data was collected non-invasively from the skin surface. Three bipolar wireless EMG (Delsys Trigno Avanti) electrodes were utilized for recording EMG signals from the skin surface above the muscle belly, away from the tendon and perpendicular to the muscle fiber. The electrodes were positioned on the dominant upper limb of the subjects. Three muscles were used for data collection: the Extensor Carpi Radialis Longus, the Palmaris Longus, and the Extensor Digitorum Muscle. The electrode placement site was first shaved to remove excessive hair and then cleaned with an alcohol swab to remove dust and dead skin particles. The excess hair on the placement site was shaved off to avoid disruptions in the collected signal.

EMG signals corresponding to four movements, hand open, hand close, wrist extension, and wrist flexion, were recorded from each subject. Eight repetitions of 5-second contractions were carried out in each session. A 5-second resting period was introduced between each contraction to prevent muscle fatigue. The muscle contraction and resting periods were displayed on the screen. Data was collected using MATLAB 2020a at a sampling rate of 2000 Hz.

**Fig. 2:**
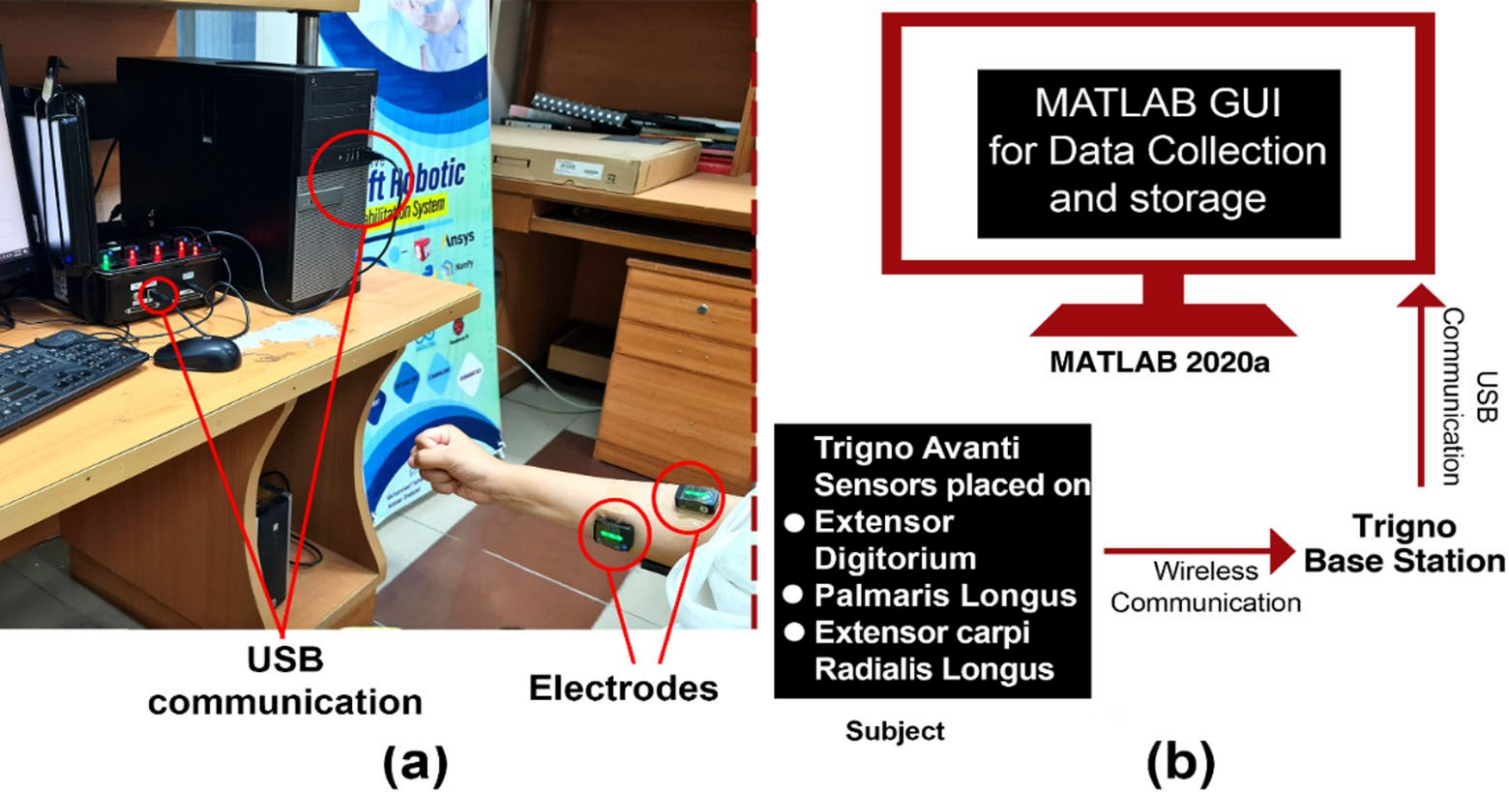
**(a)** Placement of electrodes for data collection. **(b)** Graphical illustration of the experimental setup. Three Delsys electrodes, connected to the upper limb muscles, send data wirelessly to the Trigno base station, which is then transmitted to MATLAB via USB communication.

A graphical user interface designed in MATLAB 2020a prompted each motion by displaying a selection of images on the screen, as shown in Fig. 2. Figure 2a illustrates the placement of electrodes on one subject. Figure 2b is the graphical illustration of the whole experimental procedure. The data collected from the electrodes is wirelessly transferred to the Trigno base station. The Trigno base station transfers this data to software (MATLAB 2020a) via USB communication. This data will be referred to as Dataset I throughout the rest of the paper.

To benchmark the proposed method, we have also used Ninapro datasets, which have been extensively used to assess several methods for RS^35^. The dataset Ninapro DB2^39^ was utilized along with the datasets DB3^39^ and DB8^40^. DB3 was used to validate the results for a dataset comprising amputees^39^ Table 1 gives the details of the subjects for each Ninapro dataset and dataset I. The signal corresponding to “rest” was excluded for all datasets except the Ninapro DB3 dataset.

**Table 1:** Subject information is in dataset I, Ninapro DB2, DB3, and DB8.

Datasets	No. of subjects M = male, F = female	Condition	Age
DB2	40 (28 M and 12 F)	Healthy	29.90 ± 3.90
DB3	11 (11 M)	Amputees	42.36 ± 11.96
DB8	12 (11 M and 1 F)	10 Healthy + 2 Amputees	26.5 median age
Dataset I	10 (7 M and 3 F)	Healthy	24.7 ± 2.2

### Preprocessing

The data were preprocessed and underwent two types of filters. Firstly, a 4th-order band-pass filter with a frequency range between 10 Hz and 450 Hz was applied to all the signals. The filter had an upper cutoff frequency of 450 Hz and a lower cutoff frequency of 10 Hz. After using the bandpass filter, a 2nd-order notch filter of 50 Hz was also applied to eliminate power line interference.

## Feature extraction

### Temporal features

RS performs better with temporal/time-domain features using classifiers that are better representatives of the corresponding EMG signal^13^. This is beneficial as it reduces the computation time. The primary information required for a reasonable representation based on the mathematical properties of the EMG signal can be classified into four groups: information about its complexity, information about the frequencies, prediction model approaches, and time-dependent methods. Based on these groups, the most significant features that give good accuracy are MAV for energy information, WL for complexity, and Wilson Amplitude (WAMP) for frequency information^13^. The most frequently used time-domain features, according to^11^, which provide good classification accuracy, are MAV, ZC, WL, RMS, SE, SNR, STD, and VAR. Thus, the time-domain feature vector, which was selected based on insights provided in^11,13^. It comprises eight features: MAV, ZC, WL, RMS, SE, STD, VAR, and SNR. Data segmentation was performed using a 250ms window with a 10% overlap.

### SVD-VMD features

Time-frequency analysis of the EMG signals provides both temporal and frequency-domain information. The extracted spatiotemporal information can be vital in increasing the robustness and accuracy of the RS. EMG is non-linear and non-stochastic, so we utilize VMD since it provides better decomposition of non-linear signals.

VMD is based on a generalization of the Wiener filter^26^The model estimation is variational because the estimated model and its corresponding center frequency are consistently updated. Following each estimation, the inverse Fourier transform converts the model to the time domain. The following steps involve decomposing signals into their subsequent sub-signals using the VMD method.

1. VMD splits the original signal into sub-signals called Variational Mode Functions (VMF) given by (1).

1 $$\:\text{s = }\sum\:_{\text{n=1}}^{\text{M}}{{\mu}}^{\text{n}}$$

Where s is the original signal, M is the maximum number of VMFs obtained, and n is the nth no. of VMF in the range 1 to M. $\:{\mu\:}^{n}$ is the nth sub-signal and can be defined as:

2 $$\:{{\mu}}^{\text{n}}\left(\text{t}\right)\text{= }{\text{b}}^{\text{n}}\text{(}\text{t)}\cdot\text{cos}\left({\theta}^{\text{n}}\text{(t)}\right)$$

$\:{\text{b}}^{\text{n}}\text{(t)}$ is representative of the signal envelope and $\:{\theta}^{\text{n}}\text{(t)}$ is the phase of the signal.

2. To evaluate the bandwidth of a mode, VMD involves three steps. First, for each mode $\:{{\mu}}^{\text{n}}$, the associated analytic signal is computed using the Hilbert transform, resulting in a unilateral frequency spectrum.
  3 $$\text{s}=[\delta(t)+j\pi{t}]\star(t)$$

Equation (3) exhibits a unilateral spectrum thus leading to step 2. In the second step, each mode’s frequency spectrum is shifted to “baseband” by mixing it with an exponential tuned to the estimated center frequency. Finally, the bandwidth is estimated by analyzing the Gaussian smoothness of the demodulated signal, which is the squared L^2^-norm of the gradient. These steps result in a constrained problem given in (4).

4 $$\:\left.{min}_{\left\{{u}_{n}\right\},\left\{{w}_{n}\right\}}\right|\left\{{\sum\:_{n}\parallel{\partial\:}_{t}\left[\left(\delta\:\left(t\right)+\:\frac{j}{\pi\:t}\right)\text{*}{u}_{n}\left(t\right)\right]{e}^{-j{w}_{n}t}\parallel}_{2}^{2}\right\}$$

$$\:s.t.\sum\:_{n}{u}_{n}=s$$

Where $\:{u}_{n}=\{{u}_{1},\dots\:.,{u}_{n}\}$ is the sub signals obtained from $\:s$ and $\:{w}_{n}=\{{w}_{n},\dots\:..,{w}_{n}\}$ are the center frequencies of the corresponding modes. ∑_n_ is the summation of all modes, where δ is the Dirac distribution and ∗ denotes convolution.

3. Equation (4) is a constrained problem which is changed to an unconstrained problem by the Lagrangian multiplier operator λ and the quadratic penalty factor α as given in (5). The quadratic penalty promotes reconstruction fidelity, adapting to noise levels, while the Lagrangian multiplier ensures strict constraint enforcement, combining the benefits of both approaches for optimal convergence [24].

5 $$\:L\left(\left\{{u}_{n}\right\},\left\{{w}_{n}\right\},\lambda\:\right)=\:\alpha\:\sum\:_{k}{\parallel{\partial\:}_{t}\left[\left(\delta\:\left(t\right)+\:\frac{i}{\pi\:t}\right)\text{*}{u}_{n}\left(t\right)\right]{e}^{ij{w}_{n}t}\parallel}_{2\:}^{2}$$

$$\:+\:{\parallel{s}\left(t\right)-\:\sum\:_{n}{u}_{n}\left(r\right)\parallel}_{2}^{2}+<\lambda\:\left(t\right),s\left(t\right)-\:\sum\:_{n}{u}_{n}>$$

In the above equation $\:\alpha\:$ is the regularization term which encourages the reconstruction fidelity and makes it robust to noise [24], δ is the Dirac distribution, ∗ denotes convolution, $\:{\mu\:}^{n}$ is the nth sub-signal and $\:{\partial\:}_{t}\left[\left(\delta\:\left(t\right)+\:\frac{i}{\pi\:t}\right)*{u}_{n}\left(t\right)\right]$ is the hilbert transform of $\:{u}_{n}\left(t\right)$. Transforming (5) into frequency domain gives the following expressions for $\:{u}_{n}$ and$\:\:{w}_{n}$.

6 $$\:{\widehat{u}}_{n}^{k+1}\left(w\right)=\:\frac{f\left(w\right)-\:\sum\:_{i\ne\:n}{\widehat{u}}_{i}\left(w\right)+\:\frac{\widehat{\lambda\:}\left(w\right)}{2}}{{1+2\alpha\:(w-{w}_{k})}^{2}}$$

7 $$\:{w}_{n}^{k+1}=\:\frac{{\int\:}_{0}^{{\infty\:}}w{\left|{\widehat{u}}_{n}\left(w\right)\right|}^{2}dw}{{\int\:}_{0}^{{\infty\:}}{\left|{\widehat{u}}_{n}\left(w\right)\right|}^{2}dw}$$

Each VMF produced is then segmented using a 10% overlapping window of 250ms. Since each VMF comprises several data points, a feature reduction scheme is applied to simplify the classifier. SVD is utilized to obtain singular values by taking the square roots of the obtained eigen values. The SVD of a m*n matrix can be represented as

8 $$\:\:\:\:X=US{V}^{T}$$

where $\:U$ is a m*n matrix, $\:S$ is a n*n diagonal matrix, and $\:{V}^{T}$ is also a n*n matrix. The columns of $\:U$ are the left singular vectors, and the rows of $\:{V}^{T}$are the right singular vectors. The diagonal elements of $\:S$ are nonzero and are called singular values. These values are treated as separate features, resulting in an SVD-VMD feature vector.

**Fig. 3:**
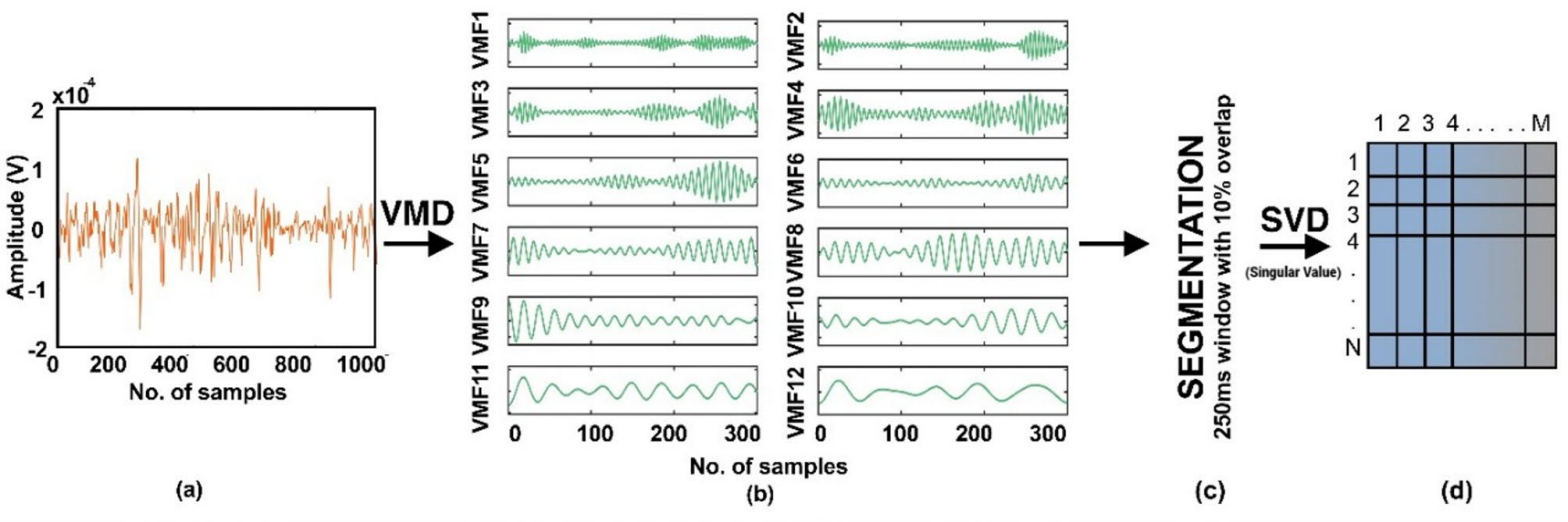
**(a)** EMG signal after pre-processing, **(b)** Decomposed variational mode functions obtained after applying variational mode decomposition, **(c)** Segmentation of obtained variational mode decomposition using an overlapping window of 250ms and 10% overlap, **(d)** Application of Singular Value Decomposition to get a singular value representation of each segment of each VMF.

Figure 3 illustrates the graphical representation of VMD-SVD feature extraction. Figure 3a shows the raw EMG signal. The signal undergoes VMD, resulting in n number of VMFs, as depicted in Fig. 3b, where *n* = 12 in this case. All the VMFs are then segmented as shown in Fig. 3c, using a window size of 250ms and a 10% overlap. Figure 3d shows the matrix n*m obtained after applying SVD.

### EMD-SVD features

EMD is a multi-resolution decomposition technique used to decompose a signal through sifting. The decomposed signals obtained through the sifting process are referred to as intrinsic mode functions (IMFs).

Each IMF is a zero-mean AM-FM signal having one extrema between zero crossings and a mean value of zero between its upper and lower envelopes. The algorithm of EMD can be described in the following steps:

1. Calculate the minima and maxima of the signal $\:f\left(t\right)$.
2. An upper $\:{e}_{u}\:\left(t\right)$ and lower $\:{e}_{l}\:\left(t\right)$ envelope is created by interpolating extracted maxima and minima using a cubic spline.
3. A middle value is calculated from the envelope $\:n\:\left(t\right)$ as $\:\:n\:\left(t\right)\:=\:{e}_{u}\:\left(t\right)\:+\:{e}_{l}\:\left(t\right)\:/\:2.$.
4. Let $\:p\:\left(t\right)\:=\:f\:\left(t\right)\:-\:n\:\left(t\right)$. If $\:p\:\left(t\right)$ is a function with zero-mean, the iteration ends, and $\:p\:\left(t\right)$ is accepted as the first IMF, i.e. $\:h\left(t\right)\:=\:p\:\left(t\right)$.
5. If not, $\:p\:\left(t\right)$ is used as the new data, and steps 1–4 are repeated, it becomes a zero-mean function.

A stopping criterion is applied to the number of sifting iterations for the IMF component to maintain its amplitude and frequency modulation. The remaining IMFs are obtained by using a sifting process to the residual signal after the first IMF, $\:h\:\left(t\right)$ has been obtained. The residual signal can be defined as in Eq. (9).

9 $$\:r\:\left(t\right)\:=\:f\:\left(t\right)-p\:\left(t\right)$$

The residual signal now contains information about the lower frequency components. The sifting procedure will be repeated until the residue reduces to either a constant, a monotonic function, or a function with only one maxima and minima. If such a signal is achieved, it will be referred to as the residue.

The decomposed signal can be represented as shown in Eq. (10).

10 $$\:f\:\left(t\right)=\:\sum\:_{\text{i=1}}^{\text{t}}{h}_{i}\left(t\right)+\:r\:\left(t\right)$$

The obtained IMFs are then segmented using an overlapping window of 250ms with a 10% overlap. The extracted VMFs have higher dimensionality, resulting in longer execution times. It was necessary to decrease this dimensionality to overcome this limitation and make the proposed algorithm suitable for real-time applications. SVD reduces the dimensionality of the resulting vector, thus reducing the execution time. Each segment obtained from segmentation using a window size of 250ms and 10% overlap then undergoes SVD to achieve a singular value representation.

### Selection of machine learning algorithms

The efficacy of the above-mentioned feature sets was evaluated for four non-parametric classifiers. Linear Discriminant Analysis (LDA) is the gold standard because of its lower execution time for RS. However, LDA performs worse than RF so it was not included in our study^41^. Moreover, authors in^42^ concluded that SVM outperforms LDA, Artificial Neural Network (ANN), and K-Nearest Neighbor (KNN). Although^42^concluded that SVM outperforms KNN^43^, used KNN to control a dexterous artificial hand. Previously, a type of Decision Tree (DT) had been used to classify wrist movements, but a comparison with other classifiers was not carried out^44^. Thus, the four classifiers chosen for our study include SVM, KNN, DT, and RF.

SVM was used with a Gaussian kernel and a box constraint set to 1 to avoid overfitting. For KNN, an exhaustive exploration of K values was conducted for each dataset, and the optimum value corresponding to each dataset was selected. An automated hyperparameter optimization procedure was executed for DT, leveraging the power of Bayesian optimization. An extensive evaluation was conducted to fine-tune the RF classifier, with tree numbers ranging from 50 to 300. Following a thorough analysis, the optimal balance between accuracy and computational efficiency was identified at 146 trees.

The dataset was balanced, with an equal number of samples for each motion; thus, the metric used to test the efficacy of the feature sets was percentage accuracy. In the 10-fold cross-validation process used for classification, the dataset was randomly partitioned into 10 subsets, or folds. During each iteration, one of these folds was used as the test set, while the remaining nine were used for training the model. This process was repeated 10 times, with a different fold designated as the test set in each iteration. The performance metrics were then averaged across all 10 folds to comprehensively evaluate the model’s generalization performance.

Accuracy = Total number of correct predictions/Total tested samples.

## Results

EMG data were collected from ten healthy subjects in the laboratory according to the experimental procedure outlined in Sect. 2.1, which is referred to as Dataset I. Dataset I was used to evaluate the performance of various feature extraction techniques and machine learning algorithms. The proposed feature extraction algorithm was also tested using publicly available Ninapro datasets.

The raw signals corresponding to all four datasets (Dataset I, Ninapro DB2, Ninapro DB3, and Ninapro DB8) were decomposed into their corresponding VMFs using VMD. A singular value representation of each decomposed VMF was obtained using SVD, resulting in a feature vector of N x M dimensionality, where N is the number of segments of the signal and M is the product of the number of channels and the number of decomposed VMFs of the signal.

Five different feature vectors were used to train each model for the dataset: Temporal feature vector (MAV, ZC, WL, RMS, VAR, and STD), EMD-SVD feature vector, VMD-SVD feature vector, EMD-SVD + temporal feature vector, and VMD-SVD + temporal feature vector. SVM, KNN, DT, and RF classifiers were employed to construct a classifier utilizing the proposed feature sets for classification. For each classifier, 10-fold cross-validation was performed to achieve robust accuracy for classification.

### Performance evaluation of VMD-SVD for dataset I

Table 2 presents the classification accuracies of different feature sets for the four classifiers (SVM, KNN, DT, and RF) on Dataset I and for the three classifiers (KNN, DT, and RF) on Ninapro DB3. Ninapro DB3 comprises 52 motions; thus, training an SVM classifier with such a high number of classes poses a significant computational expense, which is not ideal for the proposed study. The accuracies reported for Dataset I are for four motions and three channels, whereas the Ninapro DB3 dataset corresponds to 52 motions (including rest) and 12 channels. As is evident, RF outperforms all the classifiers for all the feature sets. For feature vectors VMD-SVD, the KNN classifier provides a classification accuracy higher than that of the RF classifier. For the VMD-SVD feature vector, the classification accuracy of the RF and KNN classifiers is very close, with a difference of 0.45.

**Table 2:** Classification accuracy of five feature sets (TD, EMD-SVD, VMD-SVD, TD + EMD-SVD, TD + VMD-SVD) corresponding to four different classifiers (SVM, KNN, DT, and RF) for dataset I and Ninapro DB3.

Feature vectors	Dataset	SVM (%)	KNN (%)	DT (%)	RF (%)
Time-domain	**Dataset I**	**81.83 ± 1.07**	**82.50 ± 0.4**	**87.40 ± 0.70**	**92.04 ± 0.37**
	Ninapro DB3		50.26 ± 1.25	66.32 ± 2.53	64.68 ± 1.09
EMD-SVD	**Dataset I**	**75.24 ± 1.12**	**83.02 ± 1.05**	**86.57 ± 0.80**	**90.10 ± 0.44**
	Ninapro DB3		76.71 ± 0.95	50.59 ± 1.13	61.88 ± 1.87
VMD-SVD	**Dataset I**	**86.85 ± 1.09**	**97.45 ± 0.33**	**93.37 ± 0.52**	**97.00 ± 0.21**
	Ninapro DB3		93.25 ± 0.52	89.81 ± 0.75	95.66 ± 1.30
Time-domain + EMD-SVD	**Dataset I**	**83.28 ± 1.25**	**86.66 ± 0.62**	**89.30 ± 0.61**	**92.47 ± 1.36**
	Ninapro DB3		80.41 ± 1.9	88.96 ± 0.79	86.65 ± 1.33
Time-domain + VMD-SVD	**Dataset I**	**90.35 ± 1.03**	**91.73 ± 0.66**	**92.33 ± 0.46**	**92.41 ± 0.46**
	Ninapro DB3		69.73 ± 0.80	91.38 ± 1.53	93.75 ± 0.85

A comprehensive statistical analysis was conducted to determine the significance of differences among the five feature vectors corresponding to the four classifiers. One-way Analysis of Variances (ANOVA) was employed to evaluate the overall statistical significance and the statistical significance between each group. The findings demonstrated a significant difference between the five feature sets corresponding to four classifiers (*p*-value ≤ 0.05). Multiple comparisons using Bonferroni correction resulted in a significant difference (*p*-value ≤ 0.0125) between all the groups except for the VMD-SVD feature set for KNN and RF classifier (*p*-value = 0.203) and for the VMD-SVD + Time domain set for KNN and RF (*p*-value = 0.015), RF and DT (*p*-value = 1.000). Thus, confirming that the statistical difference between the two classifiers was not significant.

**Fig. 4:**
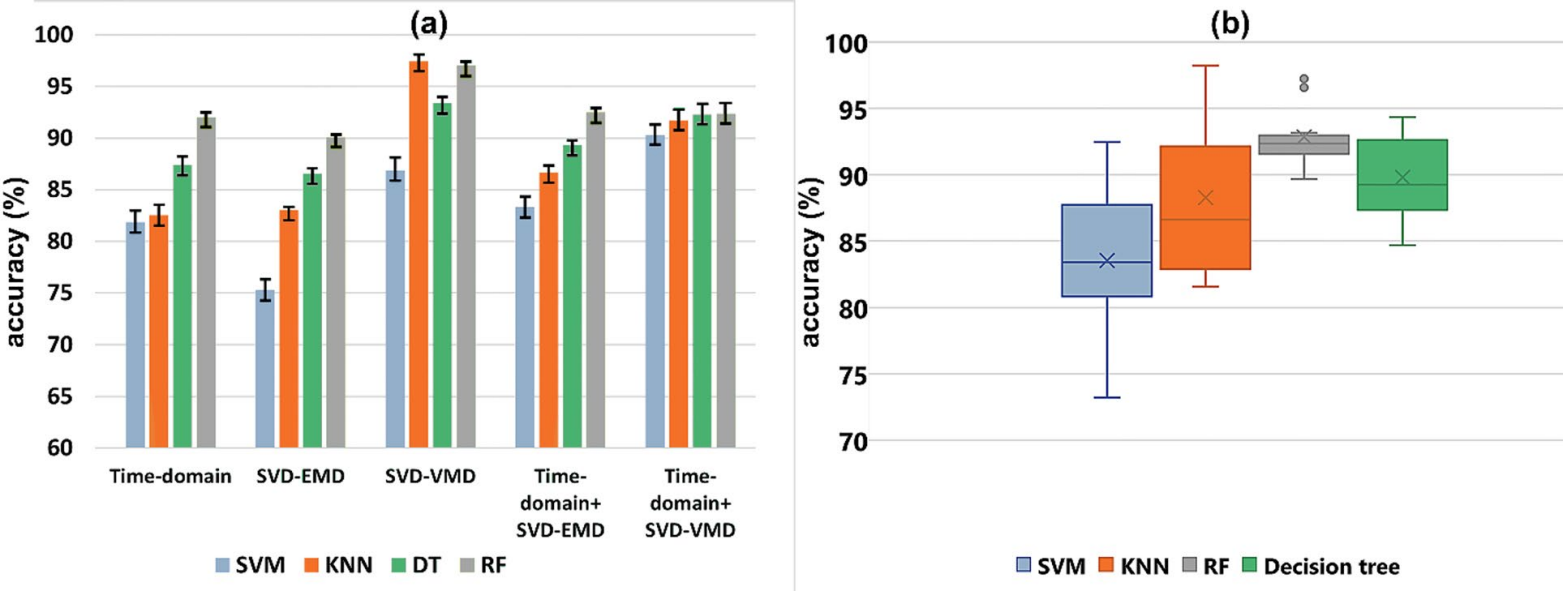
**(a)** Accuracy plot of 5 feature sets (TD, VMD-SVD, SVD-EMD, TD + VMD-SVD, and TD + EMD-SVD) for Dataset I. **(b)** Box plot depicting the mean, standard deviation, and range of accuracies corresponding to four different classifiers.

Figure 4a shows a plot of classification accuracy corresponding to five feature sets for Dataset I. As is evident, SVM provides the least classification accuracy compared to the remaining three classifiers for all the feature sets. The RF classifier, shown in green, and the KNN, in orange, perform the best among the four classifiers. Figure 4b shows a box plot comparing the accuracies of four classifiers for the five feature sets extracted. As is evident, SVM has the lowest mean and median among the four classifiers. None of the classifiers, except RF, show any outliers. However, it can be observed that the variation in accuracy obtained for RF is the least compared to other classifiers. It is also depicted that RF has the highest mean accuracy among all four classifiers.

**Fig. 5:**
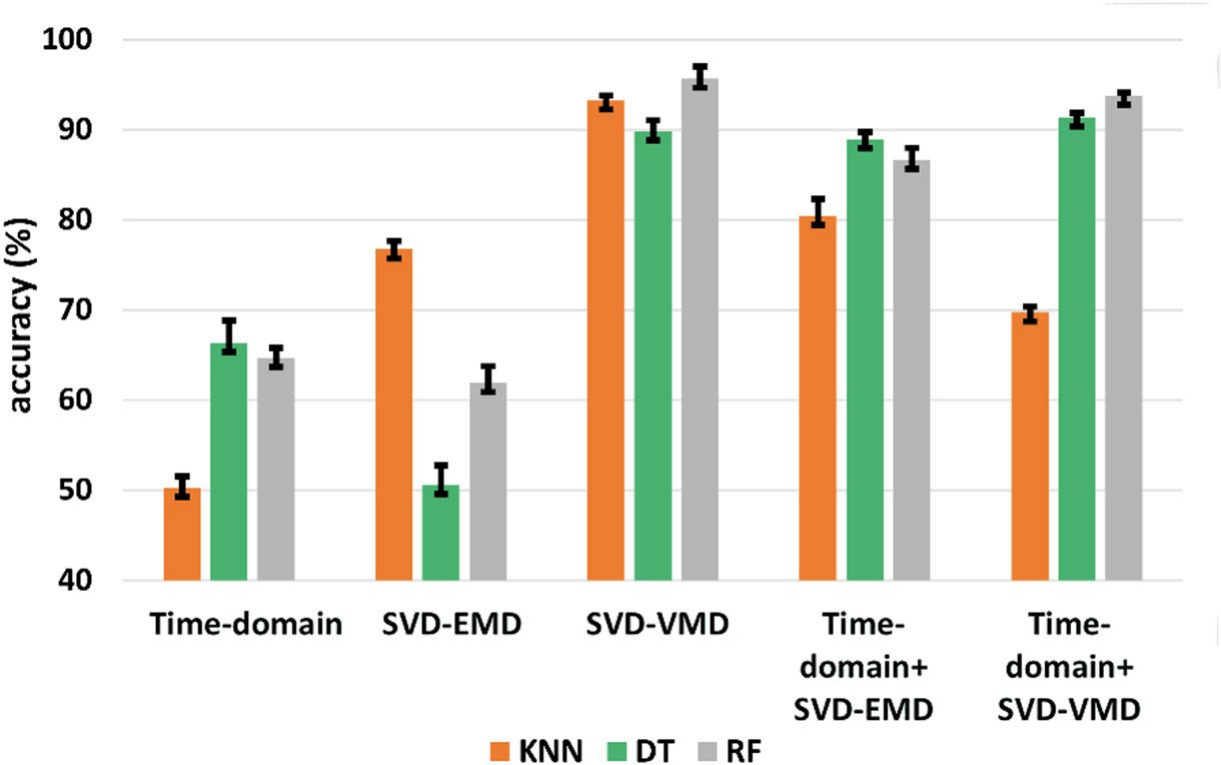
Classification accuracy of 5 feature sets corresponding to the Ninapro DB3 dataset.

Figure 5 shows the classification accuracies obtained for the Ninapro DB3 dataset for 52 motions. This dataset also comprises two amputee subjects. The proposed method outperforms all the other feature vectors corresponding to each of the classifiers.

### Efficacy of VMD-SVD for other datasets (Ninapro DB2, DB3, and DB8)

After evaluating the performance of the proposed feature extraction technique on Dataset I, it was necessary to test its efficacy for other datasets with an increased number of motions (DB2, DB3, and DB8), amputee subjects (DB3), and different protocols (DB8).

**Table 3:** Classification accuracy of the proposed feature set for publicly available Ninapro datasets (DB2, DB3, and DB8), along with dataset I, for performance evaluation of the proposed feature extraction technique.

Dataset	No. of channels	Total no. of motions	Accuracy	
			For all motions	Varied number of motions
DB2	12	49	91.43 ± 0.23	94.5 ± 0.75 for 17 motions
DB3	12	52	95.66 ± 1.67	95.12 ± 0.65 for 23 motions
DB8	16	9	98.16 ± 1.02	-
Dataset I	**3**	**4**	**97.00 ± 0.21**	**-**

**Fig. 6:**
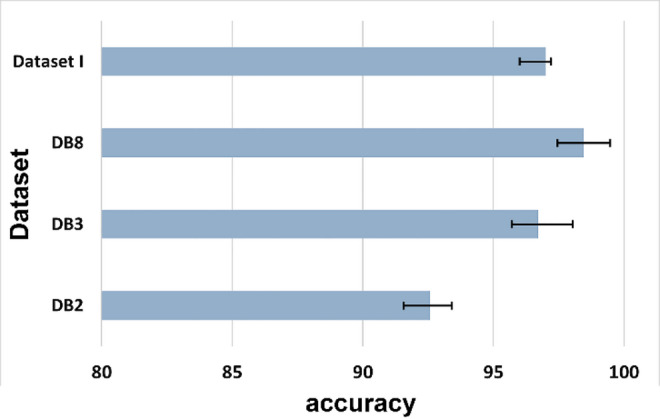
Bar chart depicting the classification accuracies of four different datasets (Dataset I, Ninapro DB2, Ninapro DB3, and Ninapro DB8) used in the study.

Table 3 presents the classification accuracies obtained using the RF classifier for the four datasets, corresponding to varying numbers of motions, i.e., 49 and 17 motions for DB2, 52 and 23 motions for DB3, 9 motions for DB8, and 4 motions for Dataset I. The proposed algorithm performs well for the DB2, DB3, and DB8 datasets. The DB2 dataset had 40 subjects, whereas DB3 comprised 11 subjects. The Coefficient of variance (COV) was computed for both datasets. DB2 had a COV of 1.79%, whereas DB3 had a COV of 0.82%. Thus, the accuracy for DB3 is higher than that of DB2 due to a smaller number of subjects and a lower COV. However, it is worth noting that the DB3 dataset contains all amputee subjects, whereas the DB2 dataset contains only healthy subjects. Although the DB8 dataset achieved the highest accuracy, it only encompassed data associated with nine motions corresponding to 12 subjects. Moreover, to evaluate the performance of the proposed method, further analysis was carried out by classifying E1 of DB2 and E2 of DB3. Figure 6 shows the classification accuracies for the four different datasets used in this study.

**Table 4:** Classification accuracy of **the** obtained results corresponds to the Ninapro DB2 dataset for varied motions (4–17) and channels (3–12).

Channels	Motions			
	4	8	13	17
3	90.27 ± 0.42	85.42 ± 0.33	79.71 ± 0.23	76.39 ± 0.26
5	95.44 ± 0.61	92.25 ± 0.37	89.29 ± 0.30	88.66 ± 0.23
8	96.62 ± 0.60	95.11 ± 0.42	93.00 ± 0.17	91.77 ± 1.58
12	**98.13 ± 0.46**	**97.80 ± 0.176**	**96.53 ± 0.12**	**95.61 ± 0.06**

Table 4 presents the classification accuracies obtained using an RF classifier for various motions and channels. Exercise B of the DB2 dataset, with 17 different isometric and isotonic motions, was selected for this analysis. The reported accuracies correspond to the RF classifier.

**Fig. 7:**
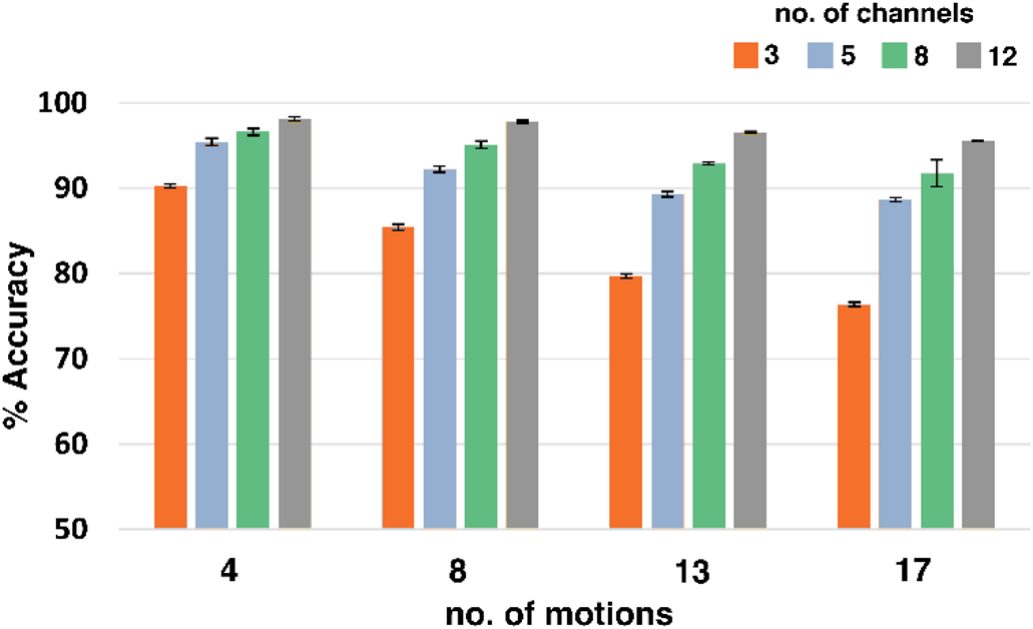
Accuracy plot of 6 feature sets extracted with varied motions (between 4 and 17) corresponding to varied no. of channels (between 3 and 12) for Ninapro DB2 dataset.

The classification accuracy of the proposed feature set for Ninapro DB2 was 90.27%, as shown in Table 3. In contrast, the dataset I obtained in the laboratory achieved an accuracy of 97% for the RF classifier, corresponding to 4 motions and 3 channels. The Ninapro DB2 dataset comprises 40 subjects, resulting in higher variance, which was tested by calculating the COV for both datasets. Subject-wise classification was performed to determine the COV. Dataset I, with 10 subjects, displayed a low COV of 0.7%, while Ninapro DB2 had a COV of 1.79%, thus explaining the difference in the accuracies of the two datasets. Moreover, the Ninapro DB2 dataset is publicly available and features fixed electrode placements; therefore, the three electrodes used for classification were chosen to be closest to the electrode placement of Dataset I. However, due to the limitation of fixed electrode positions in Ninapro DB2, slight variations from Dataset I’s electrode placement resulted in lower classification accuracies. Table 4; Fig. 7 display the classification accuracies obtained for Ninapro DB2 across various combinations of motions and channels. The motions varied between 4 and 17, whereas the channels varied between 3 and 12. It is evident from Table 4; Fig. 7 that an increase in the number of motions led to reduced classification accuracies for all channels.

### Execution time and accuracies for VMD-SVD feature vector corresponding to different numbers of VMFs

The signals were initially decomposed into 12 VMFs using VMD, resulting in a feature vector of N x 36 dimensions corresponding to four motions. To reduce the computational load of the proposed feature vector, the classification accuracy corresponding to the four classifiers was studied using six different sets of VMFs. The number of subsequent VMFs varied between 2 and 12. The number of extracted VMFs started at 2, increased by 2 in each successive set, and reached a total of 12. All the feature sets have a distinct number. VMFs were regenerated using various VMFs (2, 4, 6, 8, 10, and 12) as input from the raw EMG signals, resulting in six distinct feature sets.

Table 5 presents the results of Dataset I for each iteration, corresponding to all four classifiers. As shown in Table 5, the dimensionality of the corresponding feature vector increases with the number of decomposed VMFs. The highest accuracy corresponds to the dimensionality of Nx36, whereas the lowest accuracy corresponds to the dimensionality of Nx6. It can be observed that the feature sets corresponding to 6, 8, and 10 decomposed VMFs result in the same classification accuracy, with a minimal difference between them for the RF classifier. For the remaining three classifiers, the accuracy for 6 and 8 VMFs has a minimal difference between them.

**Table 5:** Classification accuracy and execution time of the proposed feature set with a varied number of VMFs using dataset I.

No. of VMFs	Dim. *N* = no. of segments (rows)	Execution Time(s) (2000 Hz sampling 25 s)	Accuracy (%)			
			SVM	KNN	Decision Tree	Random Forest
2	Nx6	0.31 ± 0.01	71.12 ± 2.09	89.12 ± 0.78	84.93 ± 2.85	90.34 ± 0.72
4	Nx12	0.37 ± 0.013	79.86 ± 0.76	93.71 ± 0.50	89.12 ± 0.48	93.64 ± 0.49
6	Nx18	0.50 ± 0.03	82.37 ± 0.71	95.32 ± 0.58	89.29 ± 0.51	95.13 ± 0.31
8	Nx24	0.77 ± 0.04	83.64 ± 0.93	95.74 ± 0.42	89.76 ± 0.81	94.97 ± 0.35
10	Nx30	0.91 ± 0.04	85.80 ± 0.61	96.92 ± 0.50	91.84 ± 0.48	95.51 ± 0.43
**12**	**Nx36**	**1.03 ± 0.06**	**86.8 ± 1.09**	**97.45 ± 0.33**	**93.37 ± 0.52**	**97.00 ± 0.21**

**Fig. 8:**
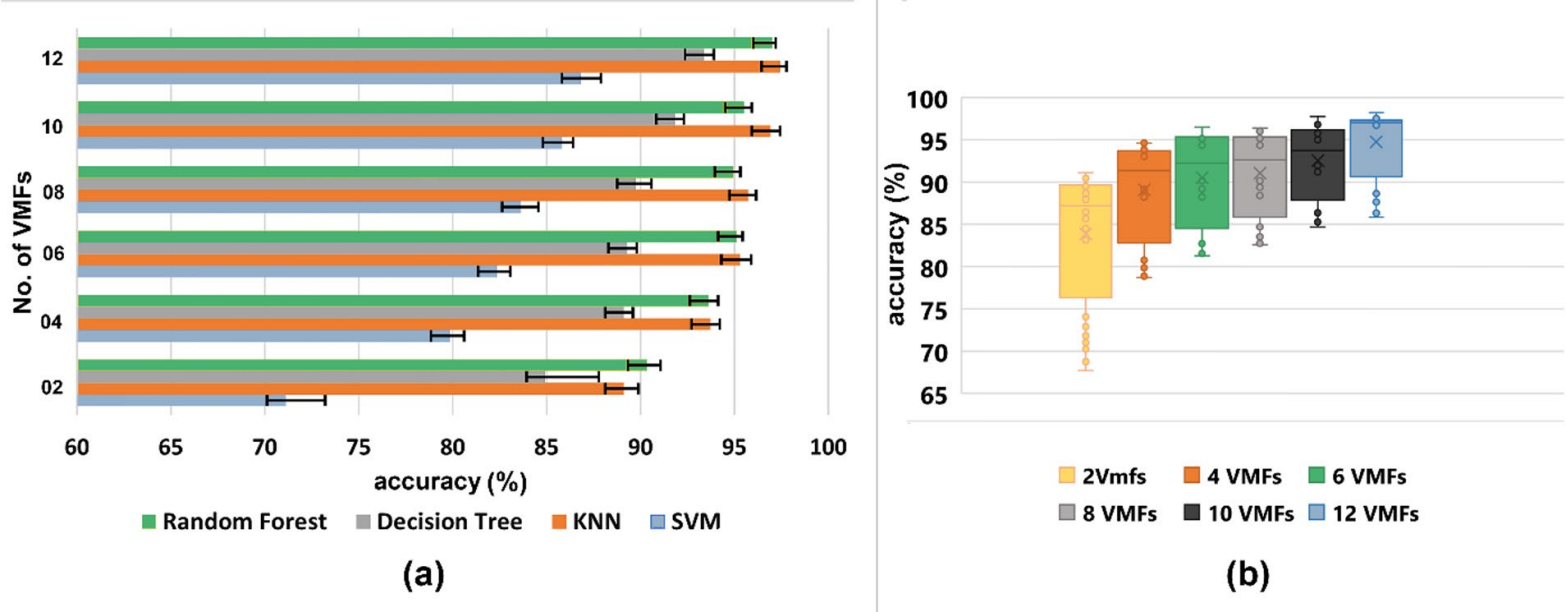
**(a)** Classification accuracies of 6 different feature sets obtained using varied VMFs corresponding to four classifiers. **(b)** Box plot depicting the mean, standard deviation, and range of accuracies corresponding to the six feature sets with varied VMFs.

Figure 8a shows the plot of accuracies for the feature vectors extracted using varied VMFs corresponding to the four classifiers used in this study. Figure 8b shows a box plot of accuracies plotted for varied numbers of VMFs in the feature sets. It is evident that the more input VMFs there are, the higher the accuracy will be. It is also depicted that the mean value corresponding to 6 and VMFs is the same, with a slight difference of 0.16. Thus, checking if the reported difference was statistically significant was crucial.

The statistical significance was assessed using one-way ANOVA, along with Tukey’s post hoc test, to determine the overall relevance and significance between each group. The analysis demonstrated an overall significance (*p*-value ≤ 0.05) among the six feature sets. Multiple comparisons between different groups showed that a significant difference exists between all the groups (*p*-value ≤ 0.05) except feature sets with 6 and 8 VMFs (*p*-value > 0.05).

**Fig. 9:**
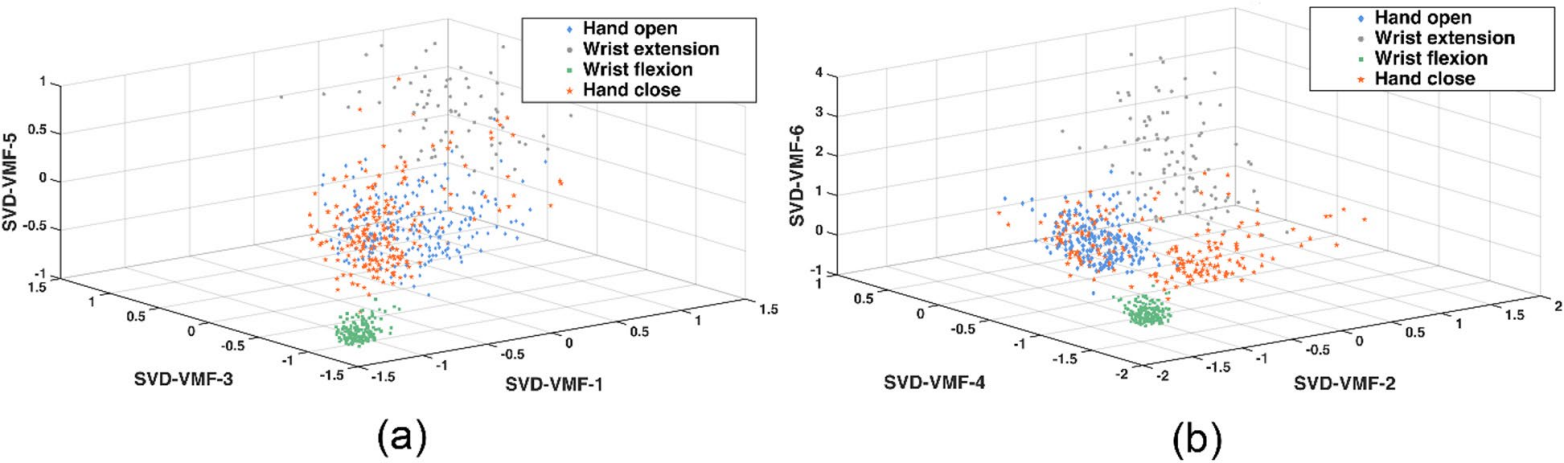
Scatter plot corresponding to (**a**) VMF-SVD1, VMF-SVD3, VMF-SVD5 features (three features selected corresponding to odd VMFs) and (**b**) VMF-SVD2, SVD-VMF4, VMF-SVD6 (three features selected corresponding to even VMFs) from VMD-SVD feature vector.

A scatter plot was plotted to visualize the distribution of some features in 3D space. Figure 9a and b show scatter plots for three features each from the proposed feature vector. It can be observed that there are overlapping regions corresponding to different motions, thereby reducing the data’s separability. This can significantly contribute to the lower accuracy obtained for SVM.

**Table 6:** Comparison of classification accuracy and execution time of the proposed feature for the optimum no. of VMFs and the TD feature vector.

Feature Vector		VMD-SVD	Time-domain
Execution time (s)		0.31 ± 0.08	0.26 ± 0.05
Dimensionality		Nx18	Nx24
**Classification accuracy** **(%)**	**KNN**	95.32 ± 0.58	82.50 ± 0.53
	**Random forest**	**95.12 ± 0.31**	**92.04 ± 0.36**

**Fig. 10:**
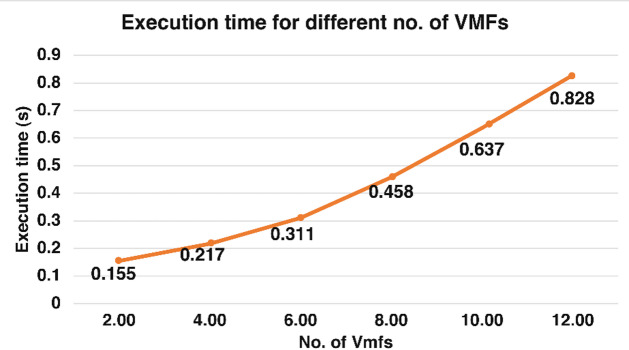
Graph showing the execution time of feature vectors obtained corresponding to various variational mode functions. The horizontal axis depicts the varying no. of VMFs, whereas the vertical axis depicts the execution time in seconds.

The execution times reported in Table 5, using different classifiers, and Table 6, using the RF classifier, were calculated for a signal of 25 s, and the processing software used was MATLAB 2020a. The PC was equipped with an i7-8550 processor and 8 GB of RAM. Figure 10 shows the execution times corresponding to different feature sets obtained with varying numbers of VMFs. It can be observed that feature vectors obtained with a lower number of VMFs have a lower execution time. However, it is also evident from Table 5; Fig. 8 that the lower number of VMFs results in lower classification accuracies.

## Discussion

EMG signals have demonstrated lower accuracy and higher sensitivity to noise in certain aspects of motion recognition, which limits their use. The use of such a system is limited to laboratory settings and cannot be implemented in a real-time environment^5^. Moreover, the previous studies utilizing VMD failed to report any analysis on amputee subjects^32,33,36^. Although the use of entropy, along with VMD, results in high accuracy^32,33^, in the presence of higher fluctuations due to noise, these methods can result in incorrect recognition^35^. SVD removes redundant and unwanted data and maps the data to a lower dimensionality^45^.

This study utilizes VMD and SVD to propose a new feature vector that provides temporal and spectral information about EMG signals, resulting in improved classification accuracy for myoelectric control systems. Before this study, the VMD-SVD-based feature extraction method had not been utilized for RS. The proposed feature extraction technique utilizes VMD to decompose the signal into various VMFs. Then, a singular value representation of each decomposed VMF is obtained using SVD. Data were decomposed into corresponding VMFs using the VMD. Each VMF contains vital spectro-temporal information regarding the signals and undergoes SVD to extract a singular value representation of this information using its eigenvalues. Four other feature vectors were also calculated to assess the efficacy of the proposed feature vector. Two of the feature sets were EMD-SVD-based and time-domain feature vectors. The remaining two combined feature vectors of the time domain are the EMD-SVD and VMD-SVD feature vectors. Five feature vectors were obtained, and their performance was evaluated corresponding to four classifiers.

As evident from Table 2, VMD-SVD results in the highest classification accuracies among all the feature sets for KNN, DT, and RF. In the case of SVM, the combined feature vector of VMD-SVD + time-domain outperforms all the other feature vectors. EMD-SVD does not perform well. The drawback of using EMD is that it results in mode mixing and is highly sensitive to noise due to its lower variance^25,46^. Although SVD is relatively stable and provides a singular value representation of the decomposed modes, due to the mode mixing and high sensitivity to noise of the EMD algorithm, the feature vector fails to perform well. VMD extracts multi-tone components and spectral bands more efficiently than EMD^47^. VMD efficiently resolves the issue of mode aliasing, resulting in significantly superior anti-noise performance and improved computational efficacy^30^. The use of a quadratic penalty in (5) preserves the original characteristics of the decomposed signal and increases the reconstruction fidelity. This results in a feature vector that is more robust and outperforms EMD-SVD. The time-domain feature vector provides only temporal information and is based on the signal’s amplitude, resulting in higher sensitivity to noise and artifacts^13^. The combined feature set, VMD-SVD + time-domain, results in higher dimensionality, and therefore, a more complex boundary will be created by the classifiers, resulting in lower classification accuracy than the individual VMD-SVD feature set.

As shown in Table 2, RF and KNN outperform other classifiers using the proposed feature vector. VMD works in both the time and frequency domains, thus resulting in a spectro-temporal feature vector that is more complex than a spectral or a temporal feature^26^. This results in a feature vector that is not easily separable and overlaps in some dimensions, as evident in Fig. 9. KNN works by finding the proximity of a data point to its corresponding neighbors, resulting in a highly convoluted separation boundary between different classes^48^. Due to these reasons, KNN contributes to a higher accuracy, as evident in Table 2. RF is an ensemble learning method that utilizes multiple decision trees, consequently performing well with EMG-based classification^49^. SVM classifies the data by creating decision planes, but as demonstrated in Fig. 9, the proposed feature vector is not easily separable, resulting in poor SVM performance. Moreover, SVM maps the data vector implicitly in the feature space, thus resulting in higher computational expense^50^. Due to the data’s non-linearity, a non-linear kernel was used, which increases the computational complexity of SVM by O(n^2^).

One key factor to consider when proposing a new algorithm for any application is its generalizability across different datasets. The use of publicly available datasets, Ninapro DB2, DB3, and DB8, has successfully proved the generalizability of the proposed feature extraction technique. It is evident from Table 3 that the proposed algorithm performed remarkably, achieving accuracies of 92.57% and 96.71%, corresponding to 49 motions for DB2 and DB3, respectively. Since DB2 comprises 40 subjects, resulting in a higher variance in the data, and DB3 contains 11 subjects, the accuracy observed for DB2 was lower than that of DB3. The DB8 dataset consisted of nine motions corresponding to 12 subjects, yielding an accuracy of 98.46%.

It is worth noting that the DB3 dataset comprises 11 amputee subjects, whereas DB8 shall consist of 2 amputees, as depicted in Table 1. As mentioned previously, the prior studies carried out did not report any analysis for the amputee subjects^31–33,35,36^. Amputee subjects have lower activation levels and are thus more susceptible to noise contamination. The proposed algorithm, however, performs exceptionally well for both healthy and amputee subjects, as depicted in Table 3.

One of the significant factors in myoelectric control systems is the execution time for feature extraction of the segment under consideration and the classifier used. KNN and RF are robust classifiers, as evident from Table 2. Although VMD has higher computational efficiency than EMD, it still has a higher execution time for feature extraction than the temporal feature vector, as it operates in both the time and frequency domains^26^. To resolve the issue of execution time, the classification performance was evaluated for different numbers of VMFs. As shown in Table 5, increasing the number of VMFs improves accuracy but also results in an undesired increase in execution time. It can also be seen that the accuracies corresponding to 6, 8, and 10 VMFs for RF are very close. The same trend for KNN corresponds to 6 and 8 VMFs. Statistical analysis revealed no significant difference between the accuracies obtained using 6 VMFs and 8 VMFs (*p*-value < 0.05).

The suggested number of VMFs for RS is 6, which represents a good tradeoff between accuracy and execution time. Table 6 summarizes the results for the performance of Dataset I, corresponding to the proposed number of VMFs for the VMD-SVD feature vector. Although the execution time is higher for the suggested VMD-SVD feature vector than for the time-domain feature vector, the classification accuracy outperforms that of the time-domain feature vector. It can also be observed that the dimensionality of the VMD-SVD feature vector is lower than that of the time-domain feature vector.

The proposed method provides a feature extraction technique that is more robust and offers higher accuracy than other methods mentioned in the study. As VMD does not have the issue of mode mixing, it results in a feature vector that is more robust to noise. Singular values are numerically more stable than eigenvalues in response to changes in the original data matrix. Thus, using singular values obtained through SVD results in features that are more robust to noise in the original signal across all movement classes. SVD addresses the variance introduced by the inherent noise in the EMG signal. To test the efficacy of the proposed method for different sets of motions, the classification accuracy was computed for a varied number of motions of the Ninapro DB2. For 4 and 17 motions, exercise set B (E2) was used, whereas for 40 motions, exercise set B (E2) as well as set C (E3) were used. As shown in Table 7, increasing the number of motions even tenfold results in only a 5% decrease in classification accuracy.

**Table 7:** A summary of classification accuracy was obtained for different numbers of classes corresponding to the Ninapro DB2 dataset.

No. of motions	Classification Accuracy
4	**98.13 ± 0.46**
17	95.61 ± 0.06
40	92.57 ± 0.07

Table 8 compares the proposed feature extraction technique with the previously reported literature utilizing VMD. It can be observed that the proposed method outperforms the previously reported results in terms of accuracy. To draw a fair comparison, we have reported the classification accuracies for a varied number of motions, which were reported in previous literature. Sharma et al.^31^., Xiao et al.^32^, Yang et al.^36^ and Wei et al.^37^, reported an accuracy of 94.28% for 6 motions, 90% for 52 motions, 97.44% for 4 motions, and 50.1% for 5 motions, respectively. Thus, we used 4, 9, 17, and 40 as standards and achieved accuracies of 98.13%, 98.16%, 95.61%, and 92.57%, respectively. Accuracy corresponding to 49 motions in DB2 and DB3 was also calculated, which was 92.43% and 96.71%, respectively.

**Table 8:** Comparison of the proposed feature extraction technique with previously reported literature utilizing VMD for RS.

	Feature extraction technique	No. of motions	% accuracy
Xiao et al.^32^	VMD + CPEI	6	94.28%
Yang et al.^36^	MVMD	52	90%
Wei et al.^37^	VMD + Entropy	4	97.44%
Sharma et al.^31^	VMD + Entropy (Utilizes ECoG signals)	5	50.1%
**Proposed method**	**VMD + SVD**	**4**	**98.13% (DB2)**
		**9**	**98.16% (DB8)**
		**17**	**95.61% (DB2)**
		**40**	**92.57% (DB2)**
		**52**	**95.66%**

Studies have shown the effectiveness of hybrid feature sets such as the Time-Domain and Power Spectral Density (TDPSD) feature set, initially introduced by Al-Timemy et al^51^., and later employed in deep learning-based models such as CNNs by Pancholi et al^52^.. Table 9 compares proposed feature vectors with previously proposed methods for RS applied to the same dataset, Ninapro DB2. As shown in Table 9, the proposed feature vector outperforms previously reported methods for classification using the same dataset.

**Table 9:** Comparison of the classification performance of the proposed method and the previous literature for Ninapro DB2.

	Classifier	Types of features	Accuracy
Atzori et al.^53^	RF	TD	75.27%
Zhai et al.^54^	CNN	Spectrogram TD	78.71%
Pancholi et al.^52^	DLPR	TD PSD	89.45%
**Proposed method**	**RF**	**VMD-SVD**	**92.66%**

Like time-domain feature extraction and EMD-based techniques, VMD also has some limitations. VMD fails to separate the DC component of the signal, resulting in a feature vector that cannot recognize inputs with high non-stationarity. This can occur due to the sudden onset of a movement^30^. Another drawback of the study is that the suggested feature vector uses SVD, which works based on variance. This can lead to discarding helpful information in cases where variance is not directly related to predictive power.

In addition to the Hudgins time-domain feature set, the new time-domain feature sets from the LibEMG open-source EMG feature extraction library, with a specific focus on LS4 (MFL, MSR, WAMP, LS), and LS9 set (MAV, ZC, SSC, WL, RMS, IAV, DASDV, VAR), have also been implemented. The performance of the LS4 and LS9 feature sets was evaluated using the same datasets (Dataset 1, DB2, DB3, DB8) and classifiers (SVM, KNN, DT, RF), as illustrated in Table 10.

**Table 10:** Classification accuracies of LS4 and LS9 feature sets across four datasets (Dataset1, DB2, DB3, DB8) using SVM, KNN, DT, and RF classifiers.

LS4				
Datasets	SVM (%)	KNN (%)	DT (%)	RF (%)
Dataset 1	82.26 ± 0.11	77.96 ± 0.12	73.47 ± 0.13	80.03 ± 0.10
DB2	30.08 ± 0.67	26.46 ± 0.04	20.05 ± 0.03	31.13 ± 0.06
DB3	16.76 ± 0.05	13.18 ± 0.05	12.66 ± 0.03	16.71 ± 0.05
DB8	52.12 ± 0.07	50.31 ± 0.06	41.66 ± 0.04	52.21 ± 0.05

LS9				
Datasets	SVM (%)	KNN (%)	DT (%)	RF (%)
Dataset 1	84.50 ± 0.13	79.82 ± 0.14	77.75 ± 0.15	82.01 ± 0.16
DB2	33.48 ± 0.73	28.56 ± 0.06	21.84 ± 0.07	35.25 ± 0.08
DB3	18.88 ± 0.07	15.28 ± 0.05	14.58 ± 0.08	20.85 ± 0.09
DB8	54.28 ± 0.09	52.47 ± 0.10	47.78 ± 0.08	57.21 ± 0.11

The proposed SVD-VMD spectro-temporal technique consistently achieves higher accuracies, thereby highlighting the robustness and superiority of the proposed spectro-temporal approach. This reinforces that while LS4 and LS9 represent an essential evolution beyond Hudgins’ features, they are still limited by their inability to capture the full non-stationary and spectro-temporal characteristics of EMG signals.

In the future, the proposed model will be deployed in real time, and the number of classification movements will be increased to achieve a robust RS for the rehabilitation of amputees and individuals with congenitally missing upper limbs. Furthermore, the aim is to investigate the effect of the regularization factor alpha for VMD, segment size, and sampling frequency on classification accuracy for the proposed feature vector.

## Conclusion

This study proposes a new spectro-temporal feature set to enhance the implementation of rehabilitation systems. Thus, the study recommends a more stable algorithm for feature extraction utilizing Singular Value Decomposition (SVD) rather than permutation entropy to address the volatile nature of entropy. Variational Mode Decomposition (VMD) and Singular Value Decomposition (SVD) were implemented, yielding a new feature vector that provides temporal and spectral information about EMG signals, resulting in improved classification accuracy for myoelectric control systems.

## Supplementary Information

Below is the link to the electronic supplementary material.

Supplementary Material 1

## Data Availability

Data sets generated during the current study are available from the corresponding author on reasonable request.
