# Supplementary material for: Enhanced spectro-temporal feature extraction for prosthetic control using variational mode decomposition
Source: Sci Rep. 2025 Nov 22;15:44386. doi: 10.1038/s41598-025-28156-6 (PMC12727768; doi:10.1038/s41598-025-28156-6)
Supplement: Supplementary file 1 — Supplementary Material 1 [file 41598_2025_28156_MOESM1_ESM.pdf]

Description of Feature sets

A. Time-domain features

The most frequently used time-domain features that provide good classification accuracy are

1. **Mean Absolute Value (MAV)** represents the average absolute value of the EMG signal, reflecting its energy.

2. **Zero Crossing (ZC)** counts the number of times the signal crosses the zero amplitude level, indicating frequency content.

3. **Waveform Length (WL)** measures the cumulative length of the waveform over the segment, indicating signal complexity.

4. **Root Mean Square (RMS)** reflects the square root of the mean power and is sensitive to contraction force.

5. **Standard Error (SE)** estimates the accuracy of the sample mean.

6. **Signal-to-Noise Ratio (SNR)** evaluates the level of signal compared to noise.

7. **Standard Deviation (STD)** indicates the dispersion of the signal from its mean.

8. **Variance (VAR)** measures the spread of the data.

B. LibEMG open-source EMG feature extraction library

1. **Maximum Fractal Length (MFL)** measures the complexity or fractal-like structure of the EMG waveform to quantify signal irregularity.

2. **Mean Square Root (MSR)** a feature related to the mean of square-rooted amplitudes.

3. **Willison Amplitude (WAMP)** Counts how many times the absolute difference between consecutive samples exceeds a predefined threshold, capturing frequency information in the time domain.

4. **L-scale (LS)** a newly proposed feature based on L-moments that capture signal characteristics in a way that’s robust to outliers.

5. **Slope Sign Changes (SSC)** counts how often the slope of the signal changes direction, reflecting frequency components.

6. **Integrated Absolute Value (IAV)** a sum of absolute signal values; represents overall signal magnitude.

7. **Difference Absolute Standard Deviation Value (DASDV)** Measures variability by looking at the standard deviation of differences between successive samples.
